# The status of e-learning, personality traits, and coping styles among medical students during the COVID-19 pandemic: a cross-sectional study

**DOI:** 10.3389/fpsyt.2023.1239583

**Published:** 2023-09-25

**Authors:** Junfan Wei, Zhengcheng Yun, Yang Zhang, Xiaoxiao Mei, Li Ba, Huan Peng, Na Li, Meng Li, Zhu Liu, Hanjiao Liu

**Affiliations:** ^1^The Seventh Clinical Medicine College of Guangzhou University of Chinese Medicine, Shenzhen, China; ^2^Southeast University School of Medicine, Nanjing, China; ^3^School of Nursing, The Hong Kong Polytechnic University, Hong Kong, China; ^4^Nursing College of Fujian, University of Traditional Chinese Medicine, Fuzhou, China; ^5^Nursing Department of The Third People's Hospital of Henan Province, Zhengzhou, China

**Keywords:** e-learning, coping styles, personality traits, medical students, COVID-19 pandemic

## Abstract

**Objective:**

The objective of this study was to explore the learning preferences and habits of medical students during the pandemic home e-learning, and to investigate the incidence of adverse emotions, optimistic character level and coping style. To explore the influencing factors of adverse emotions.

**Methods:**

A cross-sectional survey was conducted in China from March to June 2022. Medical students were recruited from three universities in China, and a questionnaire survey was conducted. The questionnaires consisted of a “e-learning preferences and habits questionnaire”, life orientation test questionnaire (LOT-R), and simple coping style questionnaire (SCSQ). Finally, a total of 492 medical students who met the inclusion and exclusion criteria became the research subjects and completed the survey.

**Results:**

A total of 57.7% believed they experienced no adverse emotions during home e-learning. ① During the COVID-19 pandemic, the score of optimistic personality of medical students was (7.25 ± 1.933), and the score of pessimistic personality was (5.82 ± 2.240). The score of positive coping was (21.75 ± 5.379), and the score of negative coping was (11.75 ± 3.611). ② The occurrence of medical students' adverse emotions during e-learning was influenced by “Whether there is a private, quiet space to study”, “Degree of knowledge mastery”, “Physical discomfort or not”, “Keep a regular schedule or not”, “Optimistic personality tendency”.

**Conclusion:**

This study demonstrates the during home e-learning, most medical students have their own learning equipment and can meet their learning needs. Their favorite mobile device to use is a mobile phone, and their favorite method of teaching is to provide course playback. More than half of medical students believe that they have some inconvenience in conducting research during home e-learning. With regard to teacher's real-time screen, the largest number of medical students support teachers turning on live screens so that they feel like they are interacting with the teacher. The preference for blended teaching is highest among medical students. In general, medical students were highly adaptive of the newest e-learning approach. Based on the statistic analysis, the factors that “Whether there is a private, quiet space to study”, “Degree of knowledge mastery”, “Physical discomfort or not”, “Keep a regular schedule or not”, and “Optimistic personality tendency” may be the influencing factors for the occurrence of adverse emotions.

## 1. Introduction

The emergence of novel coronavirus caused a pandemic that had the potential to result in infectious pneumonia that could lead to acute lung injury, acute respiratory distress syndrome and even death (1). The World Health Organization (WHO) named this coronavirus COVID-19 (2). On 30 January 2020 following the recommendations of the Emergency Committee, the WHO Director General declared that the outbreak constitutes a Public Health Emergency of International Concern (PHEIC) (3). The WHO designated COVID-19 a pandemic in March 2020 (4) [as of May 5, 2023, the WHO no longer considered COVID-19 a pandemic (5)]. In order to lower infection rates, several nations throughout the world had adopted physical quarantine measures (6–8).

In this case, the pandemic had a significant impact on traditional face-to-face education. Secretary-General of the United Nations Antonio Guterres (9) stated that as of mid-July 2020, schools in over 160 countries had closed, effecting over 1 billion students. This pervasive and persistent disruption induced by COVID-19 had resulted in a paradigm shift in the delivery of knowledge in global educational systems (10). As instruction plans were derailed (11), the graduation of some medical students was delayed and their commencements postponed (12, 13). But at the same time, the spread of the pandemic had led to the demand for medically-related talent continued to rise (14).

Consequently, some medical schools (15, 16) began to investigate network-based distance education. There were numerous instances of online learning model exploration in China (17). In this phase, various e-learning websites and platforms emerged (18), such as Massive Open Online Courses (MOOC), Superstar, Tencent and so on. This provided the necessary technical support for home e-learning. Universities were also actively exploring teaching improvement strategies to effectively improve the e-learning effect of medical students (19, 20). The benefits of this newly developed learning paradigm was that students can acquire more precise knowledge with greater efficacy, regardless of time constraints. There are also pertinent surveys indicating that students appreciate this instructional method (21, 22). On the contrary, the abrupt pandemic, the unexpected change of learning mode, and the greater learning pressure that medical students generally face may have a negative effect on their mental health (23).

Optimism is a cognitive construct. The optimistic personality trait refers to a dispositional tendency toward optimism in one's personality, which is primarily characterized by having high expectations for positive outcomes (24). Dispositional optimism is associated with better mental health (25) and physical health-related quality of life (26), better sleep quality, positive adjustment to stressful life events and coping mechanism (27, 28). Pessimistic personality traits, on the contrary, imply dispositional tendency to be more pessimistic, which is manifested by more negative expectations about the future. Different coping styles and personality traits may influence students' reactions to life events or stress, thereby influencing their psychological state, level of satisfaction, and perceptions of online learning.

This study investigated the learning status of medical students during the COVID-19 pandemic e-learning, explored the influence of personality traits and coping styles on individuals, and analyzed the influencing factors of medical students' adverse emotions. In order to understand the psychological state of medical students under major public health emergencies, and lay a foundation for the design and improvement of online learning courses for medical students and the promotion of mental health of medical students.

## 2. Objectives

The overall objective of this study was to explore the learning preferences and habits of medical students during the pandemic home e-learning, and to investigate the incidence of adverse emotions, optimistic character level and coping style. To explore the influencing factors of adverse emotions.

### 2.1. Specific objectives

1. To investigate the preferences, habits and of medical students during the COVID-19 pandemic.
2. To explore incidence of adverse emotions among medical students during the pandemic e-learning.
3. To determine the factors influencing adverse emotions among medical students during the COVID-19 pandemic with respect to e-learning.

## 3. Materials and methods

### 3.1. Study design

This study employed a cross-sectional study design. We chose to use a cross-sectional survey (29) because it is helpful for us to understand the current situation of e-learning, coping styles and personality tendencies among medical students, and to explore the influencing factors of adverse emotions. At the same time, cross-sectional research methods have been used in most studies on e-learning (30, 31) or the mental state (32, 33) of college students during the COVID-19 pandemic. An anonymous and confidential online questionnaire was administered to medical students from three universities in China. The technique of convenience sampling was utilized. Due to the difficulty of population circulation caused by the pandemic, we chose this sampling method because it has the advantages of simple operation and easy data collection, and it has been used many times in other status investigation studies (34, 35) during the COVID-19 pandemic.

### 3.2. Study subjects

This was a cross-sectional survey. The survey was conducted from March to June 2022, which came at a time when most students were conducting home e-learning due to the pandemic. The research involved three universities: Zhengzhou University, Xinxiang Medical College and Guangzhou University of Traditional Chinese Medicine.

The participants were recruited with the help of teachers and student leaders at these schools. Inclusion criteria for recruitment were as follows: (1) the student majored in medicine; (2) the student's major adopted e-learning education mode instead of traditional face-to-face school teaching; (3) the place of participation in e-learning was home; (4) participation in this study was on a voluntary basis, and (5) the student had been participating in e-learning for at least 6 months since the pandemic. Exclusion criteria: (1) students who had been diagnosed with a mental illness by a doctor before the COVID-19 pandemic; (2) those who had started their internship, stopped studying, or had not participated in e-learning.

### 3.3. Instruments

#### 3.3.1. Demographics and under environmental e-learning factors questionnaire

Author-designed, the demographics questionnaire included 12 questions. The 12 questions were gender, grade, family size, category of residence, single-child or not, “whether the e-learning caused excess expenditure,” network status, “whether there was an independent, quiet space at home to provide learning,” “whether there was a failure to attend classes on time,” knowledge mastery degree, “whether there was physical discomfort,” “whether you can keep a regular schedule”.

As for how these 12 questions were determined, we will give the following research and discussion process. First of all, gender, grade, family size, and category of residence were taken into account regarding the general characteristics of the participants. Subsequently, being a single-child in China may lead to coping poorly with stressful situations due to the country's unique policy in this regard (36). So, we thought that might be a contributing factor and asked a question related to whether or not the student was an only child. The next two questions were “network status” and “whether there was an independent, quiet space at home to provide learning”. According to social learning theory, the interaction between environment and learners affects learners' cognitive activities and explicit behaviors (37). In the online education environment, family support and technical ease of use might be two key factors (38, 39). So, we thought that these might have an impact on learning outcomes and then on mood swings, including questions concerning this. The two questions immediately following were “whether there was a failure to attend classes on time” and “knowledge mastery degree”. In the online learning environment, when exposed to specific learning tasks, learners would have a certain degree of learning anxiety (40), such as how to perform well in online learning and obtain satisfactory performance. Learning anxiety could lead to the development of adverse emotions. The last two questions were “whether there was physical discomfort” and “whether succeed in maintaining a regular schedule”. We believed that keeping a regular schedule and good physical health might be the factors affecting mental health (41). Consequently, we considered investigating these two factors with two questions.

#### 3.3.2. E-learning preference and learning habits questionnaire

The author-designed questionnaire was based on a number of interviews with medical students and relevant research (42–47). The questionnaire was designed to investigate students' habits, feelings and preferences during the e-learning. The content involved electronic product selection, teaching interaction methods preference, learning habits and other aspects. The Cronbacha's α in this investigation was 0.702.

The questionnaire was designed on the basis of small-scale interviews with medical students during e-learning and combined with relevant reference transfer. The items of the questionnaire were descriptive questions. We hoped to understand the habits and preferences of medical students during e-learning, so as to improve the satisfaction of medical students in the future. The questionnaire had good reliability, but it was not a strict scale, so the scores were not calculated nor were its items regarded as independent variables in the subsequent statistical analysis of the influencing factors of the incidence of adverse emotions. The survey results would be presented in a mainly narrative and descriptive manner, and the number of people who chose the same option for each question would be counted and expressed as a percentage.

#### 3.3.3. The life orientation test questionnaire (LOT-R)

A study conducted (48) during the COVID-19 pandemic had shown that optimism as a protective factor could reduce the occurrence of adverse emotions such as anxiety, and we believe that optimism may be an influencing factor for the occurrence of adverse emotions. Therefore, we used this questionnaire to measure the score of optimistic personality tendency of medical students. This was a self-administered scale used to test the level of optimistic personality of the participants. This questionnaire was created by psychologists Scheier and Carver (49), and consisted of ten items, each with five possible responses: “strongly disagree,” “disagree,” “uncertain,” “agree,” and “strongly agree”. Participants responded to each item with these five possible answers depending on their level of agreement with that item. For assessment, a 5-point Likert scale is utilized. The specific calculation method was that the answer “strongly disagree” was assigned a score of 0, the answer “disagree” was assigned a score of 1, the answer “uncertain” was assigned a score of 2, the answer “agree” was assigned a score of 3, and the answer “strongly agree” was assigned a score of 4.

The scale included two subscales, which were scored in the following way when calculating the respective scores of the two dimensions. Items 1, 4, and 10 reflected the dispositional optimism tendency, such that the higher the cumulative score on these three items, the more likely respondents were to exhibit optimistic personality traits. Items 3, 7, and 9 reflected the dispositional pessimism tendency, such that the greater the cumulative score on these three items, the more likely the respondent was to display pessimistic personality traits. Items 2, 5, 6, and 8 were irrelevant filling items.

When calculating the total score of this scale, the three items of pessimistic personality tendency were scored in reverse, and they were accumulated with the score of optimistic personality tendency, which was the total score of this scale.

The Chinese version was translated by Lai et al. (50) and conducted on 248 Hong Kong university students, Cronbacha's α = 0.778. In a previous study of 519 medical students studying abroad in China during the COVID-19 pandemic, the Cronbach's α of the scale was 0.71 (48). In the present study, the Cronbach's α of the scale was 0.778.

#### 3.3.4. Simplified coping style questionnaire (SCSQ)

Relevant studies (51, 52) had shown that positive coping style was beneficial to the mental health during the COVID-19 pandemic. Xie (53) adapted and translated this questionnaire into Chinese based on Folkman and Lararus' (54) Ways of Coping Questionnaire (WCQ). Xie's survey of 846 individuals demonstrated that this questionnaire was reliable and valid. In Xie's survey, Cronbacha's α = 0.899.

The questionnaire consisted of 20 items, each of which had four possible responses: “never”, “occasionally”, “sometimes”, and “often”. A four-level score of 0 to 3 Likert scale was used. Items 1 through 12 represented positive coping dimensions. The total score of these 12 items was the positive coping style score. The greater the total score, the more likely respondents were to adopt a positive coping style. Items 13 through 20 were negative coping strategies. The sum of these eight items was the total score of negative coping style. The greater the total score, the greater the likelihood that respondents would adopt a negative coping strategy. In a survey conducted by Yuan et al. (48) on medical students studying abroad in China during the COVID-19 pandemic, the Cronbach's α of the scale was 0.91. In this study, the Cronbach's α of the scale was 0.899.

### 3.4. Data collection method

The data were collected between March and June of 2022. An online questionnaire was used to capture data. Teachers and student leaders helped organize and recruit medical students, and then our team members established a Wechat group to explain the research process and purpose of this study in detail to these medical students through text notice, online meeting or telephone, and informed them that all information provided to the study would be confidential, the questionnaire would be anonymous, and they could voluntarily withdraw from the study at any time.

Then, we used a platform named “Wenjuanxing” (https://www.wjx.cn/app/survey.aspx) to distribute the formal self-administered questionnaire. The questionnaire distributed through the “Wenjuanxing” platform included two parts. The first part was an informed consent form, and the second part was a formal self-administered questionnaire. Participants had to sign the informed consent form before filling in the second part of the formal questionnaire.

Participants completed the survey anonymously to protect their confidentiality. Some questionnaires had the same answer from the beginning to the end, or the answers were inconsistent, which were considered as invalid questionnaires. All data from invalid questionnaires were eliminated from the analysis of the survey results. In this survey, 512 questionnaires were distributed, 510 were returned, and 492 were valid, for a recovery rate of 96.1%.

### 3.5. Statistical analysis

SPSS 24.0 is a professional statistical data analysis software. In previous COVID-19-related research (55) of, SPSS 24.0 had a good performance, so the data analysis of this study is conducted by SPSS 24.0. Data entry was performed using the double entry method. Firstly, descriptive statistics (56) were conducted on the demographic characteristics of the respondents, such as e-learning preferences and learning habits, as well as the adoption rate and percentage of count data. Then, the chi-square (57) was used to compare the incidence of adverse emotions under different demographic characteristics. While, the measurement data such as personality traits and coping style scores were expressed as ($\bar{x}\pm s$) (48). T test (58) was used to compare the correlation between personality trait scores, coping style scores and the incidence of adverse emotions. Finally, the significant variables in the above chi-square test, T test and Pearson correlation analysis were used as the independent variables for subsequent regression fitting, and the occurrence of adverse emotions was used as the dependent variable for collinearity diagnosis. If there was no serious multicollinearity between the independent variables, binary Logistic regression (59) was used to analyze the factors affecting adverse emotions.

## 4. Results

### 4.1. Demographic and under environmental e-learning factors data

Among the 492 participants in this survey, 180 were male, accounting for 36.6% of the total, and 312 were female, accounting for 63.4%. Less than half of the respondents in the survey were postgraduate students (43.9%), and the rest were undergraduates (56.1%). In terms of family size, more than half of the respondents had a family size of 4–5 people (56.7%), followed by about one-third with 3 or fewer people (33.5%) and the smallest number of large families with 5 or more people (9.8%). Most of the respondents lived in rural areas, accounting for nearly half of the total sample (47.2%), and the remaining respondents were divided equally between town (26.4%) and city (26.4%). A quarter (25.0%) of the respondents were the only child in their family. More than half of respondents had incurred additional expenses (55.5%) due to home e-learning during the pandemic, the remaining respondents (44.5%) did not. Most of the respondents (52.2%) had a relatively stable network in their area, followed by a very stable network (28.0%); 16.1% of the respondents had a poor network connection their area, which would affect their study; and 3.7% of the respondents had a poor network signal, which would greatly affect their use. Most of the respondents had a separate, quiet environment at home for study (75.4%), while the rest (24.6%) did not. Nearly one third (31.5%) of the respondents failed to attend class on time, while the remaining 68.5% did not. The degree of knowledge mastery of the most respondents was basically grasped (51.8%), followed by partially mastered (38.4%), and the least was not mastered (9.8%). Nearly two-thirds of the respondents (65.2%) experienced physical discomfort during home e-learning. The remaining respondents had no physical complaints. 62.4% of the respondents could keep a regular schedule, while the remaining 37.6% could not. The demographic data of the study subjects were listed in Table 1 along with the number (N) and the corresponding percentage (%).

**Table 1:** Demographic characteristics of participants.

**Demographic characteristics**	***N* (%)**	**Demographic characteristics**	***N* (%)**
Gender		Network status in the area	
Male	180 (36.6)	The network is stable	138 (28.0)
Female	312 (63.4)	The network is relatively stable	257 (52.2)
Grade		The network situation is not very good, which has an impact on learning	79 (16.1)
Undergraduate	276 (56.1)	Network signal is poor, use is very affected	18 (3.7)
Postgraduate	216 (43.9)	Whether there is a separate, quiet space at home to study	
Family size		Yes	371 (75.4)
3 people or less	165 (33.5)	No	121 (24.6)
4 to 5 people	279 (56.7)	Whether there is a failure to attend class on time	
5 or more people	48 (9.8)	Yes	155 (31.5)
Category of residence		No	337 (68.5)
Village	232 (47.2)	Degree of knowledge mastery	
Town	130 (26.4)	Basically grasped	189 (38.4)
City	130 (26.4)	Partly mastered	255 (51.8)
Single-child or not		Not mastered	48 (9.8)
Yes	123 (25.0)	Whether there is physical discomfort	
No	369 (75.0)	Yes	321 (65.2)
Whether the home e-learning caused excess expenditure		No	171 (34.8)
Yes	273 (55.5)		
No	219 (44.5)		
Whether you can keep a regular schedule			
Yes	307 (62.4)		
No	185 (37.6)		

### 4.2. E-learning preference and learning habits of medical students

In this survey, almost all respondents had their own home online learning equipment (98.6%). Regarding mobile devices used during home e-learning, mobile phone (78.9%) and laptop (78.7%) were the most frequently used, while desktop computer (7.1%) was the least frequently used. Most of the study equipment could meet the learning needs (82.7%). For the preferred teaching methods, the highest support rate was providing course playback (66.7%) and providing learning materials such as videos (63.4%), and the lowest support rate was the teacher taking roll call in class (21.1%) and asking students to turn on the camera for supervision (13.4%). As for taking notes in class, “listening to lectures and taking notes” was the most popular choice (37.8%). More than half (64.9%) of the respondents thought that there were some difficulties in scientific research during home e-learning, but it was within the acceptable range. For the view of the teacher's real-time screen, the highest support rate was to open the teacher's real-time screen so that you can feel the interaction with the teacher (32.3%). The proportion of respondents who preferred blended teaching (43.5%) was slightly higher than those who preferred face-to-face teaching (39.6%). Nearly two thirds (66.7%) of the respondents believed that home e-learning had some benefits. 56.7% of the respondents thought that they had no adverse emotions during home e-learning. And most of them (61.4%) thought that emotional problems did not affect their learning. As for the source of adverse emotions, more than one-third (33.7%) of the respondents believed it was due to their frustration regarding their difficulty in being self-disciplined when studying. For the reasons affecting the enthusiasm for class, the highest recognition rate was due to the lack of self-discipline (18.1%), followed by the lack of learning environment (15.7%). About half (49.0%) of the respondents thought that the effect of home-based learning was average. As for the main reason affecting the overall effect of home-based learning, the most recognized reason was the lack of learning atmosphere (42.1%). Other influencing factors included too many other things at home affecting study (56.5%), being affected by the Internet (23.2%), difficult to communicate with family members (22.2%), depressed atmosphere at home (16.5%) and people interrupting study (14.6%). Nearly a third of the respondents (32.3%) believed that the change in work schedule had a negative impact. The specific preferences and habits of medical students during home e-learning are shown in Table 2.

**Table 2:** E-learning preference and learning habits of medical students.

**Items**	**Statistical indicators**	***N* (%)**
Do you have your own study equipment?	Yes	485 (98.6)
	No	7 (1.4)
Which mobile devices do you use for e-learning? (multiple choice)	Laptop computer	387 (78.7)
	Desktop computer	35 (7.1)
	Tablet pc	101 (20.5)
	Mobile phone	388 (78.9)
Does your existing learning equipment meets your learning needs?	Yes	407 (82.7)
	No	85 (17.3)
Which of the following teaching methods do you prefer? (multiple choice)	Require preview before class	218 (44.3)
	Provide course playback	328 (66.7)
	Provide videos and learning materials	312 (63.4)
	Leave some homework after class	225 (45.7)
	Discussion and interaction were organized in class	174 (35.4)
	The teacher takes the roll in class	104 (21.1)
	Ask the student to open the video to see the student, so that the teacher can supervise	66 (13.4)
How do you like to take notes during online classes?	Just listen to class	91 (18.5)
	Listen to the class and take notes	186 (37.8)
	Open the textbook and listen to the class	75 (15.2)
	Listen to lectures and take notes in combination with textbooks	140 (28.5)
Do you have any research difficulties due to e-learning?	There is no inconvenience	77 (15.7)
	A little inconvenient, but acceptable	319 (64.9)
	Very inconvenient	96 (19.5)
What do you think about the real-time screen of teachers?	The real-time screen of teachers' teaching is better and has a sense of reality	121 (24.6)
	It is better to have a real-time screen of teachers teaching, and feel that they are interacting with teachers	159 (32.3)
	Whether the teacher teaching real-time screen does not matter, can hear the sound on the line	127 (25.8)
	Whether the teacher teaching real-time screen does not matter, there is a slide play on the line	83 (17.3)
Which teaching method do you prefer over classroom teaching?	Online teaching	83 (16.9)
	Face to face teaching	195 (39.6)
	Blended (online + face-to-face) teaching	214 (43.5)
Do you think there are some benefits to e-learning?	Yes	338 (66.7)
	No	154 (31.3)
Do you have adverse emotions during e-learning?	Yes	208 (42.3)
	No	284 (57.7)
Do your emotional problems interfere with learning?	Yes	190 (38.6)
	No	302 (61.4)
Where do you think your adverse emotions come from? (multiple choice)	Inability to communicate effectively with communications	92 (18.7)
	Frustration over difficulty in being self-disciplined when studying	166 (33.7)
	My study is always interfered with by my family	90 (18.3)
	Because of the pandemic, it is difficult to go out and lack of exercise leads to weight gain	103 (20.9)
	I think there are other reasons as well	28 (5.7)
What do you think is the main reason that affects your enthusiasm for class? (multiple choice)	Self-discipline is poor	89 (18.1)
	Lack of learning environment	77 (15.7)
	Teacher online course content is boring, not as good as reading books	46 (9.3)
	The network is always unstable	46 (9.3)
	Lack of textbook	48 (9.8)
What do you think of the overall effect of e-learning?	Very good	40 (8.1)
	Better	130 (26.4)
	Average	241 (49.0)
	Not good	53 (10.8)
	Extremely bad	28 (5.7)
What do you think is the main factor that affects your e-learning effect?	Anxiety about the unfolding of the pandemic	31 (6.3)
	Lack of communication with peers	32 (12.8)
	Restlessness	155 (31.5)
	Lack of learning atmosphere	207 (42.1)
	None of these conditions exist	67 (13.6)
What are the other influencing factors? (multiple choice)	Difficulties communicating with family members	109 (22.2)
	The atmosphere at home is depressing	81 (16.5)
	There are many other things at home that affect learning	278 (56.5)
	Someone at home always interferes with my study	72 (14.6)
	Learning is influenced by network stability	144 (23.2)
	The home environment is noisy	189 (38.4)
Learning is influenced by network stability	Yes	159 (32.3)
	No	333 (67.7)

### 4.3. Personality traits and coping styles of medical students

Four hundred and ninety two medical students participated in this study, and their LOT-R scores for dispositional optimism tendency were (7.25 ± 1.933) while personality pessimistic tendency were (5.82 ± 2.240), respectively. total score of optimistic personality was (13.62 ± 1.967). in the SCSQ, the positive coping style had a score of (21.75 ± 5.379) and the negative coping style had a score of (11.75 ± 3.611).

### 4.4. Effect of demographic characteristics on the incidence of adverse emotions

In this study, a total of 492 medical students were examined. Of them, 208 (42.3%) believed they experienced more unpleasant feelings while home e-learning. The findings revealed that: there were statistically significant differences in the number of family members, living environment, excess expenditure, network status in the area, whether there was a separate and quiet space at home for study, knowledge mastery, physical discomfort, and whether to maintain regular work and rest (*P < 0.05*) when comparing the general information of medical students and the occurrence of adverse emotions under the factors that may lead to adverse emotions. For information, see Table 3.

**Table 3:** Independent variables with significant results in the comparison of the incidence of adverse emotions among different demographic characteristics (*P* < 0.05).

**Items**	**Statistical indicators**	**Incidence of adverse emotions (*N*, %)**	**χ^2^**	***P***
Family size	3 people or less	56 (33.9%)	9.270	0.010
	Four to five people	125 (44.8%)		
	5 or more people	27 (56.3%)		
Category of residence	Village	111 (47.8%)	6.855	0.032
	Town	53 (40.8%)		
	City	44 (33.8%)		
Whether there is excess expenditure due to home study	Yes	134 (49.1%)	11.648	0.001
	No	74 (33.8%)		
Network status in the area.	The network is very smooth.	40 (29.0%)	27.945	0.000
	The network is relatively unobstructed, and occasionally it stalls.	113 (44.0%)		
	The network situation is not very good, which has an impact on learning.	39 (49.4%)		
	Network signal is poor, very affect the use.	16 (88.9%)		
Whether there is a separate, quiet space at home to study	Yes	128 (34.5%)	37.369	0.000
	No	80 (66.1%)		
Degree of knowledge mastery	Basically grasped	46 (24.3%)	49.307	0.000
	Partly mastered	127 (49.8%)		
	Not master	35 (72.9%)		
Whether there is physical discomfort	Yes	180 (56.1%)	72.057	0.000
	No	28 (16.4%)		
Whether a regular schedule of work and rest can be kept	Yes	85 (27.7%)	71.210	0.000
	No	123 (66.6%)		

### 4.5. Correlation analysis of personality traits, coping styles and the incidence of adverse emotions in medical students

**Figure 1:**
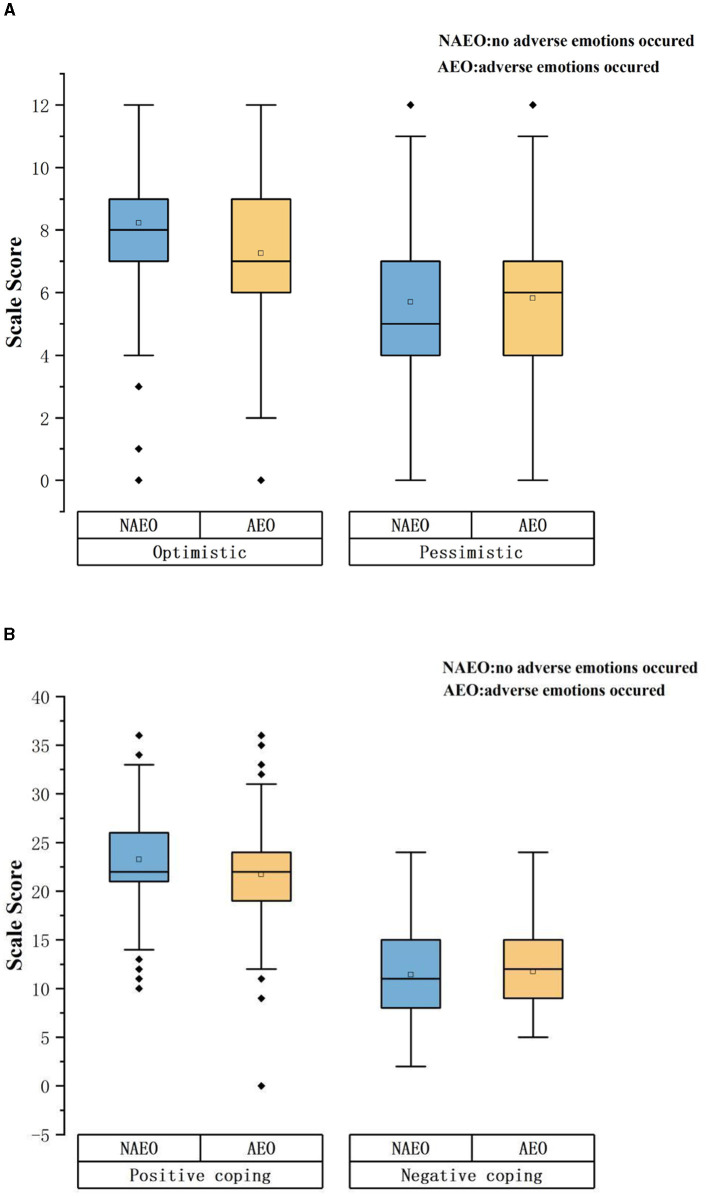
Boxplot of the personality tendencies and coping styles of medical students with different emotional conditions. **(A)** Shows the comparison of optimistic disposition score and pessimistic disposition score between medical students with and without adverse emotions. **(B)** Shows the comparison of positive coping style scores and negative coping style scores between medical students with and without adverse emotions.

There were significant differences in the scores of optimistic personality tendency and positive coping style between medical students who had experienced adverse emotions and those who had not (*P* < 0.05). Figure 1 shows a boxplot of the personality tendencies and coping styles of medical students with different emotional conditions. When the participants were divided into groups according to whether they had adverse emotions or not, there were no statistically significant differences in pessimistic personality tendency and negative coping style between different groups. See Table 4 for details.

**Table 4:** The relationship between personality traits, coping style and the presence or absence of adverse emotions.

**Scale score**	**Whether there is an adverse emotion**		**t**	***P***
	**Yes**	**No**		
Optimistic personality tendency	7.25 ± 1.933	8.22 ± 2.083	5.223	0.000
Pessimistic personality tendency	5.82 ± 2.240	5.70 ± 2.515	−0.532	0.595
Positive coping style	21.75 ± 5.379	23.26 ± 5.187	3.152	0.002
Negative coping style	11.75 ± 3.611	11.42 ± 4.179	−0.936	0.350

### 4.6. The elements that affect medical students' emotions

#### 4.6.1. Variables that were significant in univariate analysis were assigned values

Between the medical students who experienced negative feelings and those who did not (*P* < 0.05), there were significant variations in the scores of optimistic personality inclination and positive coping style. For information, see Table 5.

**Table 5:** Variables assigned for binary logistic regression.

**Index**	**Assignment**
Family size	3 or less = 1,4 to 5 persons = 2,5 or more persons = 3
Category of residence	Village = 1, town = 2, city = 3
Whether excess expenditure is incurred	Yes = 1, No = 2
Network status in the area	Very unobstructed = 1, relatively unobstructed = 2, sometimes unobstructed, sometimes stuck = 3, poor network signal = 4
Whether there is a separate, quiet learning environment	Yes = 1, no = 2
Degree of knowledge mastery	Basic mastery = 1, partial mastery = 2, and no mastery = 3
Physical discomfort	Yes = 1, no = 2
Keep a regular schedule or not	Yes = 1, no = 2
Optimistic personality tendency	Numerical variable
Positive coping style	Numerical variable

#### 4.6.2. Binary logistic regression analysis of the occurrence of adverse emotions

Variables with significant results of chi-square test, *T* test and Pearson correlation test were used as independent variables (“Family size”, “Category of residence”, “Whether excess expenditure is incurred”, “Network status in the area”, “Whether there is a separate, quiet learning environment”, “Degree of knowledge mastery”, “Physical discomfort”, “Keep a regular schedule or not”, “Optimistic personality tendency”, “Positive coping style”), and “Whether adverse emotions occurred” was used as the dependent variable. Collinearity diagnostic method was used to determine whether there was multicollinearity between independent variables. The results showed that VIF values were all <5, indicating that there was no serious multicollinearity between independent variables. Binary logistic regression could be performed.

**Figure 2:**
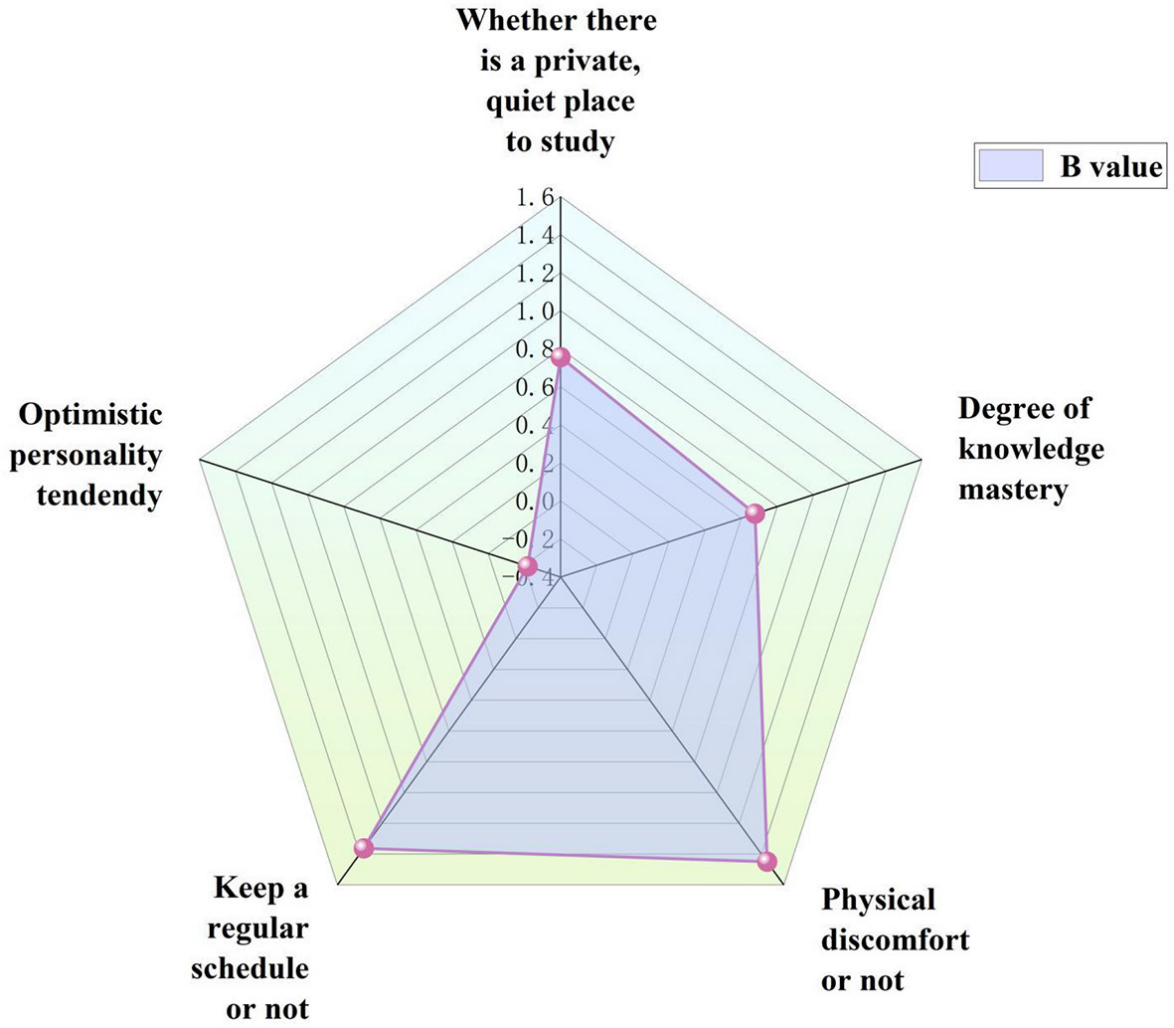
Radar chart of significant influencing factors of adverse emotions.

The significant variables in the univariate analysis were assigned in accordance with Table 5 and binary logistic regression analysis (input method) was performed with the “whether adverse emotions occurred” as the dependent variable. The outcomes revealed that the chi square value of the total model's significance test was 120.065, surpassing the significance threshold of 0.01 (*P* = 0.000), according to the results. The regression model fit was extremely good, as evidenced by the Hosmer-Lemeshow test score of 7.135, *P* = 0.522 > 0.05, which did not approach the significance threshold. Table 6 presents the binary logistic regression results. In order to make the data clear and visible, Figure 2 shows the radar chart of significant influencing factors in the regression analysis of adverse emotions. For all individuals, the percentage of correct predictions from this binary logistic regression was 74.4%. According to the results, “Whether there was a private, quiet space to study” (*P* = 0.005), “Degree of knowledge mastery” (*P* = 0.000), “Physical discomfort” (*P* = 0.000), “Keep a regular schedule or not” (*P* = 0.000) and “Optimistic personality tendency” (*P* = 0.002) were the influencing factors of adverse emotions. Tables 5, 6 include information in this regard.

**Table 6:** Results of binary logistic regression analysis of the occurrence of medical students' adverse emotions.

**Independent variable**	***B* value**	**SE**	**Walds value**	***P***	**Exp (B)**	**95%CI**
Constant	−5.841	1.105	27.952	0.000	0.003	
Family size	0.342	0.195	3.086	0.079	1.407	0.961–2.061
Category of residence	0.042	0.143	0.087	0.768	1.043	0.789–1.380
Whether excess expenditure is incurred	−0.378	0.231	2.679	0.102	0.685	0.436–1.077
Network status in the area	0.215	0.158	1.859	0.173	1.240	0.910–1.690
Whether there is a private, quiet space to study	0.756	0.269	7.901	0.005	2.130	1.257–3.610
Degree of knowledge mastery	0.677	0.190	12.706	0.000	1.967	1.356–2.854
Physical discomfort or not	1.450	0.263	30.285	0.000	4.263	2.544–7.145
Keep a regular schedule or not	1.363	0.233	34.299	0.000	3.908	2.475–6.169
Optimistic personality tendency	−0.218	0.069	9.908	0.002	0.804	0.702–0.921
Positive coping style	0.006	0.026	0.056	0.813	1.006	0.956–1.059

Cox and Snell R^2^ = 0.314, Nagelkerke R^2^ = 0.422.

## 5. Discussion

The purpose of this study was to understand the habits, preferences and adverse emotions of medical students in the pandemic period of home e-learning. To investigate the personality traits and coping styles of medical students in the pandemic period. And explored the influencing factors of medical students' adverse emotions during this period. We speculated that the occurrence of adverse emotions might be related to personality traits and coping styles. The discussions will proceed sequentially in the order of study objectives.

### 5.1. E-learning preferences and learning habits of medical students

Nearly all respondents (98.6%) had their own learning equipment. At the same time, most of the respondents (82.7%) thought that their existing learning equipment could meet their learning needs. This showed that medical students' needs for learning equipment could be basically satisfied during home e-learning.

According to the survey results, the learning tool with the highest use rate of home e-learning during home e-learning among the respondents are mobile phones (78.9%) and laptops (78.7%). This suggests that mobile phones and laptops may be the most common learning devices used by medical students during home e-learning. This might be as a result of the portability and ease of use of smartphones and laptops. In addition, more technical assistance and app development were dependent on smartphones (60). This might also be a reason for the high rate of mobile phone usage.

The highest levels of preference for a particular teaching method were for “Provide course playback” (66.7%) and “Provide videos and learning materials” (63.4%). Course replays, learning videos, and learning materials were essentially online learning resources that students can use on their own. Based on this survey, it is evident that providing accessible resources during online teaching probably was the way medical students preferred.

Based on the respondents' responses to the question “What do you think about the real-time screen of teachers?” 24.6% of the respondents to this question agreed that “The real-time screen of teachers' teaching is better and has a sense of reality”, 32.3% of the respondents believed that “It is better to have a real-time screen of teachers teaching, and feel that they are interacting with teachers”. From this we can see that more than half of the students (56.9%) wanted to be able to see a real-time video of their teacher during home e-learning. The reason is that it is more realistic or gives students a sense of interaction. This indicates that medical students prefer to be able to meet the teacher during the online teaching process, which they feel is more realistic and conducive to effective interaction. Another study (18) of online medical teaching in China during the COVID-19 pandemic also showed that interaction through online chat or video made online teaching more attractive to students.

Furthermore, the survey also revealed that only 15.7% of the respondents reported not encountering any difficulties in scientific research during the period of home e-learning. Among the remaining respondents, 64.7% believed that home e-learning presented challenges in scientific research but could be overcome, while 19.5% of the students found it highly inconvenient. This indicates that the convenience of most medical students to do research may be affected to some extent, but most students think that this inconvenience is still acceptable.

The results of the survey showed that only 16.5% of the students thought that the home e-learning mode was bad for the overall learning effect, while 68.9% of the students thought that this learning mode was beneficial in some aspects. This indicates a high acceptance of the online teaching model among students and is similar to the results of a systematic review (61). In addition, 43.5% of the students were in favor of the implementation of blended teaching methods in the future. This is similar to the research results of Yu et al. (62), that is, blended teaching has the highest support rate and significant learning effect.

The survey showed that 42.3% of the students had adverse emotions during the home e-learning process. At the same time, 61.4% of the respondents believe that emotional problems do not affect learning. This suggests that medical students were highly adaptable to home e-learning during the COVID-19 pandemic. This is contrary to the findings of a meta-analysis (63) of the prevalence of anxiety, depression, and stress in students during distance learning. This may be due to the fact that the study was conducted late in the day, and the students have largely adapted to the learning style of online instruction. It could also be because the study looked at all college students, whereas this one looked at medical students. Because of a deeper understanding of the novel coronavirus among medical students and more comprehensive knowledge of infection prevention than ordinary college students, they may be less likely to have adverse emotions such as anxiety and fear.

Among the main causes of adverse emotions considered by students, the most recognized one was difficulty in self-discipline when studying (33.7%). However, a study (64) on nursing students' adverse emotions such as anxiety and inattention during COVID-19 showed that their adverse emotions mainly came from worrying about being infected. Second was the worry of struggling to cope with school. Similar results were obtained in a separate survey (65) of students who participated in online education during the COVID-19 pandemic, which also showed that the main source of adverse emotions such as anxiety was fear of becoming infected. The reason for such different results may be due to the fact that the two surveys were conducted in the early stage of the pandemic development in this area, when our general knowledge of the novel coronavirus was not high and there was a lack of effective prevention and control methods. During the COVID-19 pandemic, pandemic prevention and control had become the norm, fear of infection, which ranked first (64) in previous studies, declined and fear of difficulty coping with school became the most important source of adverse emotions. Therefore, the main source of adverse emotions of students studying online at home at this stage was learning anxiety (40) caused by difficulty in being self-disciplined when studying. However, further studies are needed to verify this hypothesis.

Students thought that the following were the primary reasons impacting the success of their home e-learning: lack of learning atmosphere (42.1%); restlessness (31.5%); lack of communication with peers (12.8%); and anxiety about the unfolding of the pandemic (6.3%). It is clear that the major factors affecting learning are a lack of a conducive environment and restlessness. Lack of a learning environment will make students lose school supervision, become affected by the learning environment around them, even causing learners to feel lonely (66) and lower their engagement and academic achievement (67). Other influencing factors include difficulty in communicating with family members, depressed atmosphere at home, and disturbance at home, among which the most recognized is that too many things at home affect study. This is consistent with the findings of Dost et al. (68).

### 5.2. The personality traits and coping styles of medical students during the COVID-19 pandemic

The survey's findings revealed that the optimistic personality tendency score for medical students was (7.25 ± 1.933), while personality pessimistic tendency score was (5.82 ± 2.240), respectively. Total score of optimistic personality was (13.62 ± 1.967). The total score of optimistic personality was similar to Kupcewicz et al. (69) in a survey of Polish nursing students during COVID-19. The score was higher than that of Song et al. (70) in a survey of stroke patients during the COVID-19 pandemic. This result may be understandable because nursing belongs to the category of medicine, so the life orientation test questionnaire (LOT-R) scores of nursing students (69) in previous study was similar to those of medical students in our survey. It had been suggested that quality of life has an effect on optimism ratings (71), which may account for the medical students had higher LOT-R scores than stroke patients.

This study found that during the period of COVID-19, the simplified coping style questionnaire (SCSQ) score of positive coping style of medical students was (21.75 ± 5.379), and the score of negative coping style was (11.75 ± 3.611). The score of positive coping style in this study was slightly lower than the result of Li et al.'s (72) survey on 6027 college students in China. The negative coping score was substantially higher than that of other studies (33, 72) and comparable to the negative coping score obtained by Huan et al. (73) in the survey of rural inhabitants conducted for the COVID-19. The reason for this result may be due to the different time when we conducted the survey compared to the other surveys (33, 72). Our survey coincided with the second wave of COVID-19 in China. The differences in the scores of negative coping styles may be due to the different stages of the COVID-19 pandemic when the survey was conducted. This difference may also be due to the fact that the respondents and the regions where the studies were conducted were not exactly the same.

Individuals who are accustomed to adopt positive coping styles when encountering difficulties are more likely to seek solutions to problems (51, 52) and face difficulties through behavioral change or cognitive reconstruction. Therefore, college students, who tend to adopt positive coping styles, may undergo cognitive reconstruction when facing the sudden change of teaching methods, and are good at discovering some advantages of home learning that are not found in classroom teaching, such as saving travel time, replaying courses, and more diverse learning materials. They simultaneously proactively modified their approach to learning by actively embracing online learning techniques such as utilizing electronic notes, accessing online answer resources, and engaging in discussions within virtual classrooms. Through positive behavior change and psychological adjustment, these students found that home online learning was not an insurmountable problem, and thus formed positive expectations for learning effect, that is, optimistic personality tendency (24). Optimistic students tend to view the development trend of the pandemic and the effect of home-based online learning positively, thereby reducing the occurrence of adverse emotions.

Therefore, college students, who tend to adopt positive coping styles, undergo cognitive reconstruction when facing the sudden change of teaching methods, and are good at discovering some advantages of home learning that are not found in classroom teaching, such as saving travel time, replaying courses, and more diverse learning materials. Through positive behavior change and psychological adjustment, these students found that home online learning was not an insurmountable problem, and thus formed positive expectations for learning effect, that is, optimistic personality tendency (24). Optimistic students tend to view the development trend of the pandemic and the effect of home-based online learning positively, thereby reducing the occurrence of adverse emotions. Tendency and negative coping style. This is consistent with the findings of Almansa et al. (32).

### 5.3. The elements that affect medical students' emotions

According to the results of chi-square analysis, gender had no effect on the occurrence of adverse emotions. This is consistent with a study (74) conducted among college students during the COVID-19 pandemic. Meanwhile, grade had no effect, too. This was consistent with the results of another survey (48) on anxiety and depression symptoms of overseas medical students during the COVID-19 pandemic. In contrast to another study (36), the effect of being a single-child on adverse emotions status was not significant in this study. The reason for this discrepancy may be due to the different regions in which the study was conducted. Because the cultural background of each region is different, the attitude toward the only child is also different.

Although in the chi-square test, family size, category of residence, extra cost, local network status and positive coping style were the influencing factors of the occurrence of adverse emotions, but in the binary Logistic regression with the occurrence of adverse emotions as the dependent variable, the influence of these factors on the occurrence of adverse emotions became insignificant. This may be due to the interaction between these factors and other independent variables.

Regression analysis revealed that the degree of knowledge mastery, the presence of physical discomfort, the ability to maintain a regular work and rest schedule, and the availability of a separate, quiet space at home to listen to lectures, optimistic personality tendency were the main influencing factors for the occurrence of adverse emotions. This revealed that the importance of the home environment for emotional states during home-based learning. It influences students' emotions and alters their learning experiences. When learning from home, as opposed to a school library or study room, distractions such as noise, interruptions from others, and other issues might make it difficult to focus on the task at hand. This can potentially lead to feelings of anxiety and frustration.

In addition, students may underestimate their knowledge due to new teaching techniques, a lack of textbooks, and other factors, which may cause them to feel anxious, worried, and other feelings. At the same time, this is accompanied by long hours of sitting, working, studying and looking at screens. The respondents may feel physically uncomfortable or even in a state of sub-health. Many times, these bodily discomforts (75) lead to unpleasant personal experience and even create adverse emotions. Difficulty in being self-disciplined when studying, lack of supervision, or concern over the spread of the disease during home study may also prevent many students from maintaining regular work and rest schedules. Adverse emotions are frequently brought on by irregular work and rest schedules and the sleep disturbance they induce (75).

Additionally, the scores of the optimistic personality tendency and the positive coping style of the medical students with adverse emotions were significantly lower than those of the group without adverse emotions, indicating that in the face of the sudden pandemic and the change in learning style, the optimistic are more likely to see the positive side of things from the reality that cannot be changed, consider problems from a positive perspective, and find benefits.

## 6. Conclusion

In conclusion, this study demonstrates the preferences, habits among medical students during the pandemic home e-learning. The number of medical students who did not have adverse emotions was more than that of those who did, and most of them thought that their emotional problems did not affect their study, showed that medical students had a good adaptability to home e-learning during pandemic. Based on the statistic analysis, the factors that “Whether there is a private, quiet space to study”, “Degree of knowledge mastery”, “Physical discomfort or not”, “Keep a regular schedule or not”, and “Optimistic personality tendency” may be the influencing factors for the occurrence of adverse emotions.

## 7. Recommendation

This study suggests that, for the most part, the medical students studied were able to adapt well to their home e-learning. What hindered this adaptability most was unstable internet connections, a lack of self-discipline in their studies, and being without a private and quiet working environment. It is recommended that if students want to improve their experience with home e-learning they should focus on making positive changes to these hindrances. Finally, it is also important to consciously cultivate students' optimistic disposition, which may require the joint efforts of students themselves, families, schools and so on.

## 8. Limitation

In the present study, we took into account that there are many kinds of adverse emotions, especially for the participants themselves are not aware of what adverse emotions they are experiencing, but this negative experience is easy to feel. Therefore, in order to identify the medical students with adverse emotions more comprehensively, we did not use the self-rating anxiety scale or the self-rating depression scale, which is widely used in other studies (74, 76). The disadvantages of this approach are also obvious. We only know that subjects have adverse emotions during home study during the COVID-19 pandemic, but we do not know what kind of adverse emotions they are, which needs further research and a more perfect research design to verify. In addition to this, the proportion of postgraduate students in the subject group of medical students in our study is relatively high, which exceeds the proportion of postgraduate students in the normal group of medical students, which may have an impact on the results of the study, and then affect the representability of this study to the whole medical student group. Last but not least, although another study considered the cause of students' adverse emotions (64), this factor was not considered in the design of the questionnaire in this study, so it is unknown whether the bad emotions of medical students came from problems with home e-learning or with the pandemic itself; this also represents a limitation of this study.

## Data availability statement

The data that support the findings of this study are available from the corresponding author, upon reasonable request.

## Ethics statement

The studies involving humans were approved by Ethics Committee of Shenzhen Baoan District Hospital of Traditional Chinese Medicine. The studies were conducted in accordance with the local legislation and institutional requirements. The participants provided their written informed consent to participate in this study.

## Author contributions

HL, ZL, and ML were mainly responsible for the study design and guidance. JW and ZY were primarily responsible for data collection and wrote the manuscript. YZ and XM were mainly responsible for collating the data. LB, HP, and NL were mainly involved in the statistical analysis. All authors read and discussed the manuscript, reached consensus, and participated in revising the manuscript to form the final draft. All authors contributed to the article and approved the submitted version.
